# The effects of canola and olive oils consumption compared to sunflower oil, on lipid profile and hepatic steatosis in women with polycystic ovarian syndrome: a randomized controlled trial

**DOI:** 10.1186/s12944-021-01433-9

**Published:** 2021-01-29

**Authors:** Maryam Yahay, Zahra Heidari, Zahra Allameh, Reza Amani

**Affiliations:** 1 Nutrition and Food Sciences, Isfahan University of Medical Sciences, Isfahan, Iran; 2 Metabolic Liver Disease Research Center, Isfahan University of Medical Sciences, Isfahan, Iran; 3 Department of Biostatistics and Epidemiology, School of Health, Isfahan University of Medical Sciences, Isfahan, Iran; 4 Department of Obstetrics and Gynecology, Medical School, Isfahan University of Medical Sciences, Isfahan, Iran; 5 Food Security Research Center, Isfahan University of Medical Sciences, Isfahan, Iran

**Keywords:** Polycystic ovary syndrome, Canola oil, Olive oil, Lipid profile, Fatty liver, Polyunsaturated fatty acid, Monounsaturated fatty acid, HOMA-IR, SHBG

## Abstract

**Background:**

Polycystic Ovarian Syndrome (PCOS) is one of the most common endocrinopathies and metabolic disorders in women during their reproductive years. It is often associated with dyslipidemia and other risk factors of cardiovascular diseases (CVD). This study was aimed to evaluate dietary intervention effects with canola and olive oils compared to sunflower oil on lipid profile and fatty liver severity among women with PCOS.

**Method:**

This study was a 10-week intervention including 72 women with PCOS. Patients were randomly assigned to three groups for receiving 25 g/day canola, olive, or sunflower oils for 10 weeks. The primary and secondary outcomes were to assess changes in lipid profile and in fatty liver severity, respectively.

**Result:**

At the end of the study, 72 patients with a mean age of 29.31 were analysed. Canola oil consumption resulted in a significant reduction in serum levels of TG (*P* = 0.002) and TC/HDL (*P* = 0.021), LDL/HDL (*P* = 0.047), and TG/HDL (*P* = 0.001) ratios, however, there was no significant reduction in lipid profile following olive oil consumption. Canola (*P* < 0.001) and olive oils (*P* = 0.005) could significantly reduce the fatty liver grade. Moreover, HOMA-IR in both canola (*P* < 0.001) and olive (*P* = 0.004) groups was significantly decreased.

**Conclusion:**

In total, compared to olive and sunflower oils, significant improvements in lipid profile, liver function, and HOMA-IR were observed following canola oil consumption in women with PCOS.

**Trial registration:**

IR.MUI.RESEARCH.REC.1397.315. Registered 30 JUNE 2019 - Retrospectively registered, https://www.irct.ir/trial/38684

## Introduction

Polycystic Ovarian Syndrome (PCOS) is one of the most common endocrinopathies and metabolic disorders in women during their reproductive years [1, 2]. Regarding the study of Ding et al. (2017), in women with reproductive age, the PCOS prevalence was estimated from 5 to 18% which is higher in obese and diabetic women [3]. Considering Rotterdam criteria, this syndrome is characterized by symptoms such as hirsutism, oligomenorrhea, infertility, obesity, anovulation, and acne [4]. The syndrome’s etiology is not precisely known, and researchers suggest a combination of genetic and environmental factors. Insulin Resistance (IR) is a typical characteristic of this syndrome which is found in 25–60% of women with PCOS [5]. One of the factors involved in PCOS etiology is oxidative stress which increases the levels of free radicals and results in elevated lipid peroxidation and insulin resistance [6]. On the other hand, various studies showed that women with polycystic ovary syndrome are at greater risk for chronic diseases, especially cardiovascular disease (CVD) [7–9]. Moreover, PCOS increases the risk of dyslipidemia. Dyslipidemia in PCOS is associated with prevalent insulin resistance, although, not all women with PCOS have IR [10]. Considering previous studies, dyslipidemia in PCOS has a multifactorial nature [6, 11]. In a meta-analysis, researchers reported that women with PCOS have 26 mg/dL higher triglycerides, lower HDL-cholesterol, and higher non-HDL-cholesterol levels compared with their non-PCOS counterparts [12]. Nutritional management has a key role in obesity, hepatic steatosis, and other metabolic pathologies [13]. Studies showed that dietary fatty acid patterns could exert important effects on PCOS related complications, including obesity, imbalanced glycemic and hormonal homeostasis, inflammation, and dysregulation of adipokine production [14, 15]. Replacing saturated fatty acids (SFAs) with polyunsaturated fatty acids (PUFAs) can reduce insulin resistance resulting in improved lipid profile [16, 17]. Moreover, PUFAs consumption has various beneficial effects on chronic disorders [18, 19]. Hence, omega-3 PUFAs, as mainly eicosapentaenoic acid (EPA) and docosahexaenoic acid (DHA) are substrates for signaling molecules regulating the liver lipid metabolism and enhanced the lipid profile [20, 21]. This feature is achieved through (i) transcriptional activation of FA oxidation by peroxisome proliferator-activated receptor alpha (PPAR-α); and (ii) suppression of de novo lipogenesis via down-regulation of sterol regulatory element-binding protein 1 (SREBP-1c) [13, 22]. Some studies showed that women can significantly convert alpha-linolenic acid (ALA) into EPA and DHA compared to men, with estimated net conversion rates of ALA to EPA of 21% vs. 8% and of ALA to DHA of 9% vs. 0%, respectively [23, 24]. Women also have a higher concentration of serum DHA [25]. Besides, decreased n6/n3 PUFA ratio may be correlated to an augment of Δ5- and Δ6-desaturase activity and consequently more conversion of ALA to omega-3 fatty acids, especially EPA [26, 27].

Canola oil is a rich source of PUFAs, especially linoleic acid, alpha-linolenic acid (ALA), and mono-unsaturated fatty acids (MUFAs) [28]. Olive oil is one of the Mediterranean diet main components that is rich in MUFAs and dietary polyphenols [29]. Although canola and olive oils have higher MUFAs, ALA is higher, and n6/n3 PUFA ratio is lower in canola oil; therefore, it appears to be a healthier choice [30].

Previous studies showed positive effects of olive and canola oils in dyslipidemia modulation [31–33]. Moreover, dietary patterns with a higher amount of PUFAs and MUFAs have beneficial effects including antiatherogenic and anti-inflammatory actions which can improve non-alcoholic fatty liver disease (NAFLD) severity [34, 35].

Homeostasis model evaluation for insulin resistance index (HOMA-IR) is the most highly utilized marker among static IR indices as a surrogate measurement of IR in large population researches. Thus, studies surveying the prevalence of IR in PCOS are greatly inconsistent mainly based on different methods applied and cut-offs chosen to define IR [36–38]. Furthermore, an ongoing debate is present whether IR in PCOS is due to obesity alone or obesity aggravates IR intrinsic to PCOS [39, 40].

Sex hormone-binding globulin (SHBG) is expressed in the human liver under controlling the hormones and nutritional factors [41]. The human liver secretes SHBG into the blood, where it binds to androgens and estrogens with high affinity, regulating their bioavailability. BMI is considered the main determinant of circulating SHBG concentrations, and a negative correlation between BMI and SHBG plasma levels was consistently reported [42].

Therefore, liver fat content rather than total body or visceral fat has been reported as the main determinant of circulating SHBG [43, 44]. Low serum SHBG concentrations in overweight individuals are a biomarker for metabolic syndrome [45, 46] and are predictive of type 2 diabetes (T2D) [47, 48] and CVD risk [49–51].

Furthermore, SHBG plays a central role in PCOS pathophysiology [52]. SHBG binds to testosterone with high affinity, hence, regulating free testosterone levels [53]. Circulating SHBG concentrations are characteristically low in patients with PCOS. These women have elevated androgen levels in which IR is present. Compensatory hyperinsulinemia and IR inhibit the hepatic synthesis and secretion of SHBG [54]. Hyperinsulinemia can increase androgen and free androgen production by reducing androgen binding with SHBG. Overall, these hormonal imbalances can progress to metabolic and cardiovascular diseases [55].

Despite the significant role of edible oils on lipid profile and considering significant discrepancies in research findings there is no clinical trial that has compared the effects of canola and olive oils on PCOS women. Due to the appropriate fatty acid content of canola and olive oils and since sunflower oil is one of the most common and cheapest edible oils in most areas, the present study was designed to evaluate the effects of these three oils on lipid profile, fatty liver, HOMA-IR and SHBG in women with PCOS.

## Materials and methods

### Study design and patients’ characteristics

This study was conducted as a randomized, double-blind, controlled clinical trial that conformed to Helsinki’s declaration and Good Clinical Practice Guidelines. The study’s protocol was reviewed and approved by Medical Ethics Committee at the Isfahan University of the Medical Sciences, Iran (ethics registration number: IR.MUI.RESEARCH.REC.1397.315) which was registered at the Iranian Registry of Clinical Trials (approval code: 38684). The participants included 90 patients with PCOS aged 18 to 45 years. Eligible patients were those with PCOS who were referred to gynecology clinics in Isfahan. Participants were included based on Rotterdam criteria [56] in which two following features were confirmed: 1) Oligomenorrhea (the interval between two menstrual periods more than 35 days) or amenorrhea (no vaginal bleeding for at least 6 months); 2) Clinical findings of increased blood androgen levels (hirsutism scores greater than 7 or obvious acne), or increased blood testosterone levels (testosterone levels above 2 nmol/L) and 3) Polycystic ovaries in ultrasound scan (12 follicles measuring 2–9 mm in diameter, or ovarian volume > 10 mL in at least one ovary). Patients were eligible if they had not taken any fat-lowering or omega-3 supplements in the last 3 months. Furthermore, if they were not menopausal, and not following a special diet, having BMI between 25 and < 40, and not having a severe weight loss history in the last 6 months. Participants were excluded if they had the following conditions: pregnancy, adrenal hyperplasia, androgen-secreting tumors, Cushing syndrome, hyperprolactinemia, thyroid dysfunction, diabetes or other metabolic diseases, using lipid-lowering medications, tamoxifen, raloxifene, oral or injectable corticosteroids, any history of intolerance to the canola and olive oils. Moreover, patients who underwent chemotherapy and smoked were excluded. All participants were given the necessary explanations regarding the study protocol, and they completed written informed consent forms before commencing the study.

### Sample size

The sample size was calculated based on the standard formula for clinical trials, regarding the type 1 error (α) of .05 and type 2 error (β) of 10 (power = 90%) considering serum TG levels in the study of Salar et al. [57]. Accordingly, 27 subjects were calculated for each treatment group. Considering three probable dropouts in each group, the final sample size of 30 individuals in each group was obtained.

### Fatty acid compositions of the oils

Fatty Acids (FA) composition of refined olive, canola, and sunflower oils (Oila, Isfahan, Iran) were evaluated at the reference food chemistry laboratory (Meyar Danesh Pars laboratory, Isfahan, Iran). FA composition of the three oils was determined using high-performance gas chromatography [57]. Olive oil contained 68.93% oleic acid as the main FA while canola oil contained 59.62% as oleic acid and 7.37% as ALA. Sunflower oil contained 60.51% linoleic acid as its main FA. Also, the n-6/n-3 ratio was 184 for sunflower oil, 17.8 for olive oil, and 2.9 for canola oil (Table 1).

**Table 1:** Fatty acid composition of three oils studied^a^

Variable		Sunflower oil	Canola oil	Olive oil
SFA (%)	Palmitic Acid	6.75	5.55	13.78
	Stearic Acid	3.18	2.42	3.09
	TOTAL	11.39	9.26	17.55
N-9 MUFA (%)	Oleic Acid	27.52	59.62	68.93
	TOTAL	27.70	61.39	69.22
N-6 PUFA (%)	Linoleic Acid	60.51	21.21	11.13
	TOTAL	60.71	21.31	11.20
N-3 PUFA (%)	Linolenic Acid	0.09	7.23	0.63
	TOTAL	0.33	7.37	0.63
n-6/n-3 PUFA		183.96	2.89	17.77

^a^*MUFA* Monounsaturated Fatty Acid, *SFA* saturated fatty acid

### Randomization and intervention

Patients were randomly assigned to the three equal groups to receive either 25 g canola or olive oils as the main intervention groups and the control group consumed 25 g sunflower oil daily for 10 weeks. The random allocation was done by an investigator who was not directly involved in the trial. Participants were given the oils in similar bottles. Neither participants nor the researchers and physicians were aware of the type of oils consumed to the end of the study. Bottles with the oils were given to the women for the first 5 weeks at the beginning of the study, and it was repeated in the next 5 weeks. Also, a small container was provided which exactly contained 25 g to pour the oil they needed every day. Women were asked to add oil to their salads or meals after cooking (e.g., rice, stew, etc.) so that the oil was not exposed to heat. All patients were advised to take a balanced diet with a macronutrient distribution of 45–60% as carbohydrate, 30 to 35% as fat, and 15–18% as protein. The diet was described and each woman was given an exchange list to ensure diet consistency during the 10-week intervention. Subjects were advised to limit fish and nuts intake (walnuts and fish at most once a week), and avoid taking omega-3 or flaxseed supplements. Also, all patients were asked not to change their physical activity patterns through the study. Participants were followed via short text messages and phone calls to detect any possible adverse effects. The compliance rate was determined by the number of empty bottles returned; those who consumed 85% of oils or more were considered as an adherent to the study.

### Anthropometric data and dietary intake

The weight and body composition of the participants were evaluated by portable TANITA M780 (Tanita, Japan), and the height was measured by Seca 763 scale (Hamburg, Germany). Without shoes in a standing position, the weight was measured with light clothes. Height was measured in a standing position, looking straight ahead, arms at sides, and shoulders relaxed with no shoes on with 0.1 cm precision. Then, BMI was calculated by dividing the weight (kg) by the square of height (m^2^). A trained nutritionist evaluated the participants’ dietary intake by a three-day food record questionnaire. Patients were asked to complete three-day food record questionnaires (two consecutive days and a day-off) at the beginning and end of the study. Then, all food items were entered into a customized Nutritionist IV software (1997, First Databank Inc., San Bruno, CA), and mean intake of energy, macro and micronutrients were calculated at the baseline and 10-week post-intervention.

### Physical activity assessment

Participants filled in a validated form of the 7-item International Physical Activity Questionnaire (IPAQ) pre-and post-intervention. Data were converted to metabolic equivalent hour/week [58].

### Laboratory data

The blood samples were obtained from all participants after 12 h of fasting. Blood samples were centrifuged at 3000 rpm for 10 min. Separated serums were frozen at − 80 °C until further biochemical measurements. Serum concentrations of TG, HDL-c, LDL-c, and total cholesterol were measured using an enzymatic colorimetric method (Pars Azmoon, Tehran, Iran). Non-HDL cholesterol was calculated by subtracting HDL-c from TC and represented LDL-c + IDL-c + VLDL-c cholesterol fractions [59]. The SHBG levels were measured using an enzyme-linked immunosorbent assay (IMMULITE 2000 SHBG).

At the beginning and end of the study, liver sonography was performed by a skilled radiologist for all patients using an ultrasound device (General Electric LOGIQ E9- using probe 3.5/5 MHz, USA). The radiologist was blind to the study process and study groups. A rating method was introduced to achieve a semi-quantitative measure of the extent of fat deposition in the liver. Based on liver echo-texture parameters, the brightness of the liver, the contrast ratio of liver-to-kidney, and blurred vessels, the degree of hepatic fatty infiltration was scored from grades 0 to 3.

Fasting serum glucose (FSG) and serum insulin (FSI) were measured using spectrophotometry and radioimmunoassay (IMMULITE 2000 Insulin), respectively. Then, HOMA-IR was calculated using the following formula for each participant [60, 61]:

HOMA-IR = (FSG × FSI) / 405.

### Statistical analysis

SPSS software version 21 (IBM Corp. IBM SPSS Statistics for Windows, Armonk, NY) was used to analyze data. Kolmogorov–Smirnov test evaluated the normality quantitative data. Data were presented as mean ± standard deviation (SD) for quantitative variables and frequency (%) for categorical variables. A Chi-square test was applied to analyze qualitative variables. The changes of quantitative variables were compared pre-and post-intervention using a paired sample t-test. The baseline characteristics of participants were compared between the groups by independent samples t-test or Chi-Square test. One-way analysis of variance (ANOVA) and LSD post-hoc tests was used to compare the groups in terms of quantitative variables. Also, the covariance analysis (ANCOVA) was used to adjust the effect of confounding variables. *P*-value < 0.05 was statistically considered significant.

## Results

### Study baseline characteristics

**Fig. 1:**
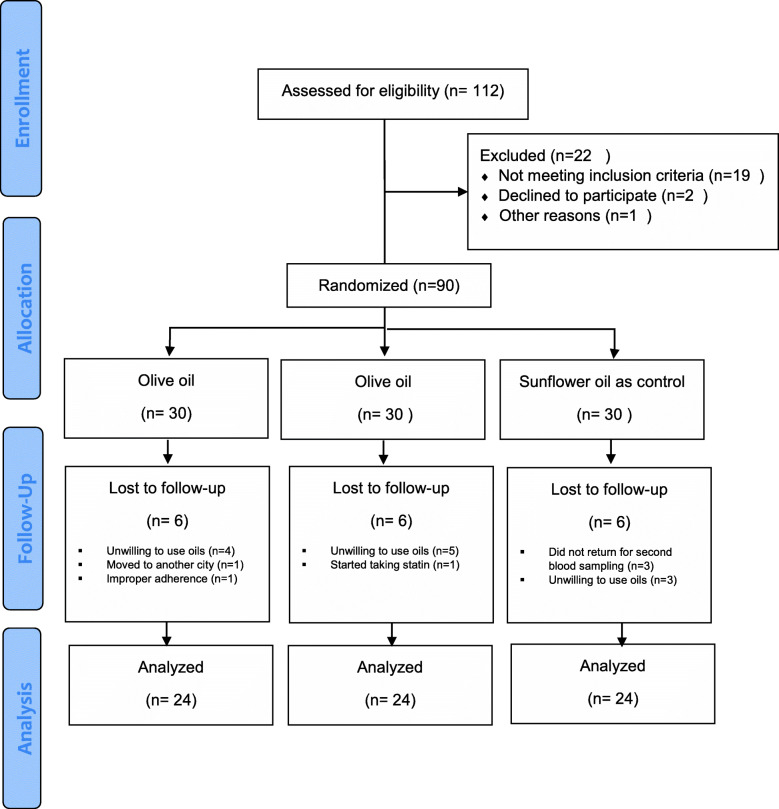
Flow chart of patient recruitment for the clinical trial

Totally, 72 participants (80%) with a mean age of 29.31 ± 6.52 completed the trial. Eighteen participants (six in each group) failed to follow the protocol (Fig. 1). The participants’ baseline characteristics are presented in Table 2. There were no significant differences between the three groups in age, height, weight, disease duration, physical activity, marital status, taking OCP, and metformin at the baseline and last 3 months.

**Table 2:** Baseline characteristics of the participants ^a^

variable		sunflower oil group (*n* = 24)	canola oil group (*n* = 24)	olive oil group (n = 24)	*P*_value^*^
Age (years)		28.08 (6.17)	31.75 (6.35)	29.58 (7.26)	0.163
Weight (kg)		80.07 (12.97)	71.95 (7.14)	73.97 (12.47)	0.038
BMI (kg/m^2^)		29.47 (3.57)	27.74 (1.91)	28.84 (4.44)	0.127
Duration of PCOS (years)		2 [1–4.74]	2 [0.31–9.5]	5 [1–9]	0.434
Physical Activity (Met-Hours/wk)		19.69 (23.54)	17.37 (24.97)	14.17 (16.27)	0.689
Marital	Single	5 (20.8%)	2 (8.3%)	4 (16.7%)	0.472
	married	19 (79.2%)	22 (91.7%)	20 (83.3%)	
OCP prescription (last 3 months)	YES	5 (20.8%)	5 (20.8%)	5 (20.8%)	1.00
	NO	19 (79.2%)	19 (79.2%)	19 (79.2%)	
Metformin prescription (last 3 months)	YES	2 (8.3%)	2 (8.3%)	3 (12.5%)	0.85
	NO	22 (91.7%)	22 (91.7%)	21 (87.5%)	

*OCP* Oral contraceptive pill, *PCOS* Polycystic ovary syndrome

^a^Values are mean (SD) or Median [Q1-Q3] or Number (percent)

^*^ *P*-values are resulted from ANOVA or Chi-Square or kruskal-wallis test

### Dietary intake of participants

Table 3 summarizes the macronutrients and micronutrients intake of participants. Statistical analysis of energy, macronutrients, and dietary intake of PUFA, SFA, LA, and dietary fiber showed no significant differences between the three groups at baseline and end of the study. However, at the end of the study, the dietary intake of MUFA was significantly higher in canola and olive oils groups (*P* < 0.001). Also, dietary intake of ALA was significantly increased in the canola oil group (*P* < 0.001).

**Table 3:** Comparison of dietary intake between the groups at the baseline and following intervention^a^

Variable	sunflower oil group(*n* = 24)				canola oil group(n = 24)				olive oil group(n = 24)				P-value**
	before	after	Change	*P*-value*	before	after	change	*P*-value*	before	after	change	*P*-value*	
Energy (kcal)	2056.62 (390.05)	1883.73 (278.78)	− 172.89	.013	2184.57 (276.26)	1947.40 (357.62)	− 237.17	.001	2076.89 (352.01)	1951.6 (325.45)	− 125.24	.000	0.947
Carbohydrate (g)	323.44 (78.88)	262.73 (60.07)	−60.71	.003	297.70 (58.49)	238.49 (81.75)	−59.21	.001	312.85 (62.54)	287.81 (54.40)	−25.04	.035	0.077
Protein (g)	75.83 (19.99)	79.33 (25.16)	3.50	.528	77.84 (26.95)	83.12 (18.54)	5.28	.264	73.32 (12.66)	81.86 (15.07)	8.54	.010	0.771
Fat (g)	55.35 (22.18)	66.82 (25.43)	11.46	.086	62.96 (19.42)	82.63 (24.00)	19.67	.002	60.08 (16.89)	73.51 (26.65)	13.42	.006	0.318
SFA (g)	18.36 3.67))	17.83 (5.34)	−0.53	.643	20.53 (5.45)	16.49 (5.02)	−4.03	.014	16.55 (4.57)	18.88 (6.02)	2.32	.018	0.275
PUFA (g)	18.36 7.31))	18.46 (7.43)	0.10	.875	22.26 (5.07)	19.59 (5.56)	−2.67	.003	20.87 (6.93)	18.94 (5.90)	−1.92	.023	0.089
MUFA(g)	13.47 4.87))	13.19 (4.41)	−0.27	.644	13.74 (4.26)	18.91 (3.39)	5.17	.000	14.71 (3.21)	18.004 (2.86)	3.28	.000	< 0.001
LA(g)	16.40 9.59))	16.03 (8.24)	−0.37	.854	21.85 (7.72)	17.58 (7.11)	−4.26	.016	19.04 (11.59)	15.87 (7.05)	−3.17	.145	0.816
ALA (g)	.130 .09)0)	0.13 (0.08)	0.002	.932	0.14 (0.08)	0.27 (0.07)	0.13	.000	0.14 (0.07)	0.14 (0.13)	0.005	.851	0.000
Dietary fiber(g)	18.48 6.76))	18.57 (5.81)	0.10	.928	16.90 (6.08)	17.52 (5.53)	0.61	.431	18.92 (4.98)	19.75 (7.05)	0.82	.549	0.812

^a^*ALA* Alpha-linolenic acid, *LA* Linoleic acid, *SFA* saturated fatty acid, *PUFA* Polyunsaturated fatty acid, *MUFA* Monounsaturated fatty acid

*P-values are resulted from paired samples t-test. **P-values are resulted from ANCOVA, adjusted for baseline

### The effect of canola and olive oil on lipid profile

Table 4 compares the lipid profile, HOMA-IR, and SHBG among canola, olive, and sunflower groups at the baseline and following 10 weeks of the study. At the beginning of the study, no significant differences were observed among the three groups in terms of lipid profile variables. After 10 weeks of intervention, there was a significant reduction in serum TG (*P* = 0.012), non-HDL-c (*P* = 0.014), TC/HDL-c (*P* = 0.007), LDL-c/HDL-c (*P* = 0.040), and TG/HDL-c (*P* = 0.006) in the canola group. HOMA-IR in the canola group (*P < 0.001*) and in the olive group (*P = 0.004*) significantly declined. Also, a significant marginal drop in serum TC (*P* = 0.076) and LDL-c (*P* = 0.055) levels in canola oil was seen at the end. Canola oil supplementation caused a significant reduction in serum levels of TG (*P* = 0.004), and TC/HDL-c (*P* = 0.021), LDL-c/HDL-c (*P* = 0.047), TG/HDL-c (*P* = 0.001) and HOMA-IR (*P < 0.001*) indices compared to olive and sunflower oils. The changes were significant after adjusting the confounding factors (Table 4).

**Table 4:** Comparison of serum lipid profile, HOMA-IR and SHBG between the groups at the baseline and following intervention ^a^

Variable^b^		Sunflower (*n* = 24)	canola (*n* = 24)	Olive (*n* = 24)	P^*^	*P*- adjusted^**^
TG (mg/dL)	Baseline	140.08 (81.04)	140.38 (42.22)	121.78 (64.93)	0.37	0.66
	10th wk.	143.37 (64.46)	134.33 (33.09)	122.61 (59.66)	0.03	0.04
	Changes	3.29	−6.08	0.82	0.004	0.002
	P^***^	0.112	0.012	0.522		
TC (mg/dL)	Baseline	177.29 (38.31)	188.50 (34.23)	180.21 (31.89)	0.136	0.265
	10th wk.	178.20 (39.45)	175.46 (35.98)	177.79 (29.27)	0.35	0.412
	Changes	0.91	−13.04	−2.41	0.876	0.91
	P^***^	0.83	0.076	0.778		
LDL (mg/dL)	Baseline	109.21 (32.59)	121.71 (29.45)	116.35 (27.56)	0.32	0.47
	10th wk.	114.46 (32.24)	109.63 (31.54)	111.48 (23.63)	0.512	0.563
	Changes	5.25	−12.08	−4.87	0.276	0.36
	P^***^	0.203	0.055	0.387		
HDL (mg/dL)	Baseline	40.83 (6.96)	40.68 (9.35)	39.82 (9.18)	0.22	0.26
	10th wk.	40.29 (7.70)	41.27 (9.09)	39.39 (9.45)	0.49	0.52
	Changes	−0.54	1.37	−0.75	0.353	0.391
	P^***^	0.168	0.425	0.38		
Non-HDL (mg/dL)	Baseline	136.46 (42.24)	148.29 (31.09)	140.26 (33.65)	0.64	0.94
	10th wk.	137.92 (43.05)	133.88 (33.42)	135.70 (29.82)	0.32	0.46
	Changes	1.45	−14.41	−4.56	0.144	0.239
	P^***^	0. 735	0.014	0. 99		
TC/HDL	Baseline	4.59 (1.82)	4.85 (0.97)	4.59 (1.29)	0.07	0.16
	10th wk	4.74 (2.18)	4.34 (1.03)	4.53 (1.19)	< 0.001	< 0.001
	Changes	0.15	−0.51	−0.06	0.021	0.04
	P^***^	0.397	0.007	0.765		
LDL/HDL	Baseline	2.72 (1.13)	3.13 (0.82)	2.94 (0.93)	0.89	0.89
	10th wk	2.83 (1.08)	2.70 (0.85)	2.89 (0.88)	0.05	0.007
	Changes	0.11	−0.43	−0.05	0.047	0.049
	P^***^	0.116	0. 040	0. 696		
TG/HDL	Baseline	3.84 (3.29)	3.75 (1.66)	3.27 (2.38)	0.343	0.176
	10th wk	3.99 (3.00)	2.97 (1.27)	3.33 (2.25)	0.350	0.06
	Changes	0.15	−0.78	0.06	0.001	0.001
	P^***^	0.06	0. 006	.526		
HOMA-IR	Baseline	3.01 (1.92)	2.71 (1.58)	2.30 (0.86)	0.351	0.345
	10th wk	3.46 (2.35)	1.90 (1.33)	1.73 (0.95)	0.001	0.001
	Changes	0.45	−0.81	−0.57	< 0.001	0.002
	P^***^	0.201	< 0.001	0.004		
SHBG (nmol/L)	Baseline	50.96 (34.84)	32.47 (14.11)	44.06 (35.69)	0.112	0.030
	10th wk	42.50 (22.49)	38.07 (22.9)	45.62 (37.57)	0.703	0.503
	Changes	−8.46	5.6	1.56	0.410	0.139
	P^***^	0.124	0.161	0.836		

^a^ *HDL* high-density lipoproteins, *LDL* low-density lipoproteins, *TG* triglyceride, *TC* Total cholesterol, *HOMA-IR* Homeostatic Model Assessment of Insulin Resistance, *SHBG* Sex hormone-binding globulin. ^b^ Data are presented as mean (SD) or geometric mean (SD). ^*^ Calculated using one-way ANOVA. ^**^ Calculated using ANCOVA, adjusted for the effect of energy, carbohydrate, protein, weight and baselines. ^***^ Calculated using paired sample t-test

Within- and between-group changes in fatty liver severity are summarized in Table 5. In within-group comparisons, fatty liver severity was significantly reduced in canola (*P* < 0.001) and olive (*P* = 0.005) groups, while no changes in the control group were evident. Moreover, there were no significant differences between canola and olive oil groups regarding fatty liver severity reduction (*P* = 0.404). Furthermore, the SHBG levels did not change significantly in all groups post-intervention.

**Table 5:** Comparisons of the Changes of fatty Liver Status from Baseline to the End of the Intervention in the three Groups

Grade of NAFLD %	sunflower oil group(n = 24)			canola oil group(n = 24)			olive oil group(n = 24)			*P*-value
	before	after	*P*-value	before	after	*P*-value	before	after	*P*-value	
Grade 0	11 (45.8)	11 (45.8)	1	6 (25.0)	16 (66.7)	< 0.001	10 (41.7)	17 (70.8)	0.005	0.404
Grade 1	9 (37.5)	9 (37.5)		13 (54.2)	6 (25.0)		12 (50.0)	6 (25.0)		
Grade 2	4 (16.7)	4 (16.7)		5 (20.8)	2 (8.3)		2 (8.3)	1 s(4.2)		

*NAFLD* Non-Alcoholic Fatty Liver Disease

*P*-values are resulted from ANOVA or Chi-Square or kruskal-wallis test

## Discussion

The present study investigated the effects of canola, olive, and sunflower oils on lipid profile and NAFLD severity among women with PCOS for 70 days. Findings showed that canola oil can exert beneficial effects on some lipid profile parameters and fatty liver severity. Moreover, olive oil consumption resulted in significant improvement in fatty liver severity, while it had no effects on the lipid profile.

Canola and olive oils compared to sunflower oil, caused a marked decrease in HOMA-IR. However, SHBG levels had no significant changes in all three groups post-intervention. Our results were similar to the findings of some studies which reported improved insulin sensitivity by consuming omega-3 fatty acids. In 2012, Rafraf et al. conducted a study on 61 overweight or obese women with PCOS and reported significant reductions in FSG, FSI, and HOMA-IR but no significant changes in weight, BMI after taking omega-3 fatty acids [62]. Omega-3 fatty acids could exert their positive effect on insulin sensitivity by altering glucose transporters (GLUT1 and GLUT4) levels in muscle and adipose tissue. Moreover, Nigam et al. showed improved insulin sensitivity in NAFLD subjects using high-MUFA oil compared to a control oil group. The proposed mechanism can be the improvement of glucagon-like peptide-1 responses in insulin-resistant individuals and up-regulation of glucose transporter-2 expression in the liver [32]. Conversely, other studies presented no significant differences in BMI, FSG, FSI, HOMA-IR, SHBG after receiving 4 g of omega-3 fatty acids per day [63, 64].

Previous studies reported that the changes in dietary fatty acid composition, especially replacement of SFA with PUFA and MUFA can improve blood lipid levels even in those participants with low initial concentrations [65]. Participants in the canola oil group demonstrated a significant decrease in serum TG and Non-HDL-c concentrations and TC/HDL-c, LDL-c/HDL-c, and TG/HDL-c ratios. Also, a marginally significant effect on TC and LDL-c concentrations was observed in canola oil. Ghobadi et al., in their meta-analysis study, showed that canola oil decreased LDL-c and TC compared to sunflower oil [66, 67]. Also, Sarkkinen et al. performed a 6-month dietary intervention trial in hypercholesterolemic patients and found that LDL-c levels in the canola oil group were lowered by 3.7% from the baseline levels [68]. Canola oil’s effect on TC and LDL-c may depend on the types of fatty acids replaced by oil, study duration, and baseline levels of TC and LDL-c [69].

Looking at the previous findings, the effects of various oils on blood lipids is a controversial area [70, 71]. Although olive and canola oils contain high amounts of MUFA [72], canola oil has a higher content of PUFAs, especially ALA, and lower SFA [28]. The exact mechanisms of Canola oil on serum lipids are not well known; however, it could be related to its fatty acid composition [73]. Furthermore, in this trial, canola oil compared to olive oil caused more reduction in HOMA-IR (0.81 vs. 0.57; Table 4). Higher ALA intake can elevate insulin secretion, improve insulin sensitivity, and lipoprotein lipase activity, leading to serum TG reduction [74]. After consuming canola oil, this can be the reason for the significant decrease in TG concentrations. Olive oil consumption led to a non-significant increase in TG levels. Moreover, omega-3 FA content of canola oil can down-regulate VLDL-c and apolipoprotein-B100 synthesis which can, in turn, lower serum TG concentrations [12]. Previous studies reported that a dietary pattern with a higher PUFA amount could decrease serum LDL-c and TC but not TG and HDL-c [75, 76]. Canola oil supplementation caused a marginally significant reduction in LDL-c and TC concentrations (Table 4). Salar et al. in a clinical trial showed that canola oil at the dose of 30 g/day decreased serum LDL-c more than sunflower oil, a poor source of ALA [57]. Furthermore, in Sacks et al.’s trial, they found a significant decrement in serum LDL-c and TC/HDL-c ratio following the replacement of SFA by canola oil [77]. In addition, canola oil consumption led to a significant reduction in TC/HDL-c, LDL-c /HDL-c, and TG/HDL-c ratios, known as the main predictors of CVD [78]. Higher intake of canola oil can reduce the risk of coronary heart disease and all-cause and cardiovascular mortality attributed to its PUFA content [73].

This intervention was unable to find any significant reduction in lipid profile in response to olive oil consumption. The results of some previous studies are in contradiction with available findings. Venturini et al., in their clinical trial, found that extra virgin 10 mL/day olive oil combined with fish oil (3 g/day) lowered the serum levels of TC and TC/HDL-c and LDL-c/HDL-c ratios [33]. Extra virgin olive oil in a dosage of 4 g/day in mildly hypocholesterolemic participants caused favorable changes in plasma lipid profile [79]. The mechanisms in which olive oil can exert its beneficial antioxidative effect can be clarified by polyphenol activity or through the cumulative protective effect of its polyphenols and MUFA content [78]. In a recent meta-analysis, researchers reported that olive oil significantly reduced serum levels of TC, LDL-c, and TG but to less extent than other vegetable oils, specifically in subgroup analysis for refined olive oil [80]. In this research, using refined olive oil could cause a lack of beneficial effects of olive oil on blood lipids. Also, different FA compositions of olive oil compared to canola oil could be a reason for the impaired beneficial effect on lipid profile (Table 1). Ghobadi et al. demonstrated that the reduction of TC by olive oil was significantly lower than that of omega-3 rich oils [80]. On the other hand, the ratio of n-6 to n-3 fatty acids is known to influence inflammatory state in PCOS women [81]. Several studies illustrated that a higher intake of n-6 PUFA increases CVD risk, which is independently associated with elevated serum TC and TG levels [82].

Olive and canola oils could decrease fatty liver severity; however, they had no significant differences. Nigam et al. in an interventional study including 93 males with NAFLD, showed that consuming olive and canola oils at the dose of 20 g/day resulted in a significant decline in fatty liver grade and other NAFLD risk factors [32]. Kruse et al. compared the efficacy of canola and olive oils on hepatic steatosis in obese men. In their study, 27 obese men consumed 50 g/day of either canola or olive oils for 8 weeks. The results showed that canola oil compared to olive oil caused a greater reduction in hepatic steatosis [81].

Dietary ingredients, especially the type and amount of fats, are important in liver fat deposition, which is responsible for 15% of liver fat content [31]. Reduction in n-3 PUFA levels and augment in n-6 PUFAs exacerbate hepatic steatosis by inhibiting transcription factor activity of PPAR-α which promotes a pro-lipogenic and pro-inflammatory state. PPAR-α is presented in tissues with a high FA oxidation activity, such as the liver [83–85]. The faster oxidation of MUFAs can clarify the positive effects of olive oil on hepatic fat content than SFA in the postprandial phase [86]. For instance, consumption of diets rich in MUFA (28% of total calories) for 8 weeks by patients with type 2 diabetes reduced liver fat by 30%. This decline was associated with elevated postprandial β oxidation of fatty acids [87]. Errazuriz et al. in their RCT indicated that high MUFA intake (28% of energy, half as olive oil) in prediabetes patients for 12 weeks lowered hepatic fat and improved insulin sensitivity. Moreover, a high-MUFA diet boosts lipoprotein lipase activity more than a diet rich in SFA resulting in enhanced clearance of circulating triglyceride-rich lipoproteins [86]. Furthermore, a higher amount of MUFA and ALA in canola oil can exert beneficial effects against fatty liver by improving insulin sensitivity, glucagon-like peptide-1 responses, and up-regulation of glucose transporter-2 expression in the liver of insulin-resistant participants [88]. In addition, a higher intake of ALA and a lower ratio of n6/n3 PUFA can enhance lipid oxidation, inhibit hepatic triacylglycerol synthesis by PPAR-α activation mechanism. This transcription factor precisely plays a role in lipid metabolism by favoring the expression of numerous genes involved in FA oxidation, which inhibit de novo lipogenesis [89–91]. Therefore, PPAR-α activation is likely to improve NAFLD [92].

In this study, only women were included. There is a sex difference in the conversion of ALA to EPA and DHA, which is higher in women than that of men. The females have higher erythrocyte phospholipid EPA and higher plasma DHA content. Previous studies showed an inverse relationship between the menopausal status of women and the age of female participants with the change in plasma EPA content after olive oil supplementation [24, 25]. A part of this study inconsistency compared to other studies could be attributed to such differences.

SHBG expression regulation by oleic acid can be modulated or even attenuated by estradiol. Moreover, olive oil intake is associated with elevated levels of SHBG and PPAR- downregulation induced by oleoyl-CoA that seems to be an underlying mechanism in such regulation. Interestingly, olive oil is one of the main components of the Mediterranean diet and SHBG levels were associated with a lower risk of cardiovascular disease [93]. To add more, Oner and Muderris carried out a study on 45 non-obese women with PCOS. They received 1500 mg of omega-3 fatty acids daily for 6 months. Findings showed a significant decrease in BMI, FSI, HOMA-IR and a significant increase in SHBG and TNF-α due to supplementation with omega-3 fatty acids [94]. The limitations of the present study can be the cause of insignificant effect at the SHBG level.

No significant results were found for sunflower oil effect on lipid profile and fatty liver severity. Sunflower oil is a rich source of omega-6 fatty acids. Previous studies indicated that high intake of foods rich in omega-6 fatty acids could exacerbate inflammation and liver damage [84, 95, 96]. It is worthy to note that due to the availability and cost of sunflower oil compared to olive or canola oil, most people, especially middle-income or low-income families tend to use this type of oil in their food preparation.

### Study strength and limitation

The study design was a double-blind randomized clinical trial to assess three types of conventional oils from different sources while comparing two high MUFA oils. The main limitation of this study was the method of fatty liver evaluation. Ultrasound is not very accurate to detect mild cases of the fatty liver compared to the more recent Fibroscan method. The other limitation was the study duration. It was not possible to measure the gene which promotes the SHBG and also to measure the ALA, EPA, and DHA content of erythrocyte membranes as a good indicator of the participants’ adherence.

## Conclusion

Canola oil, as a good source of MUFA and ALA, showed beneficial effects on lipid profile compared to olive, and sunflower oils in women with PCOS. The role of ALA and a lower ratio of n6/n3 PUFA should be considered in addition to the positive effect of MUFA in the canola oil. Also, the substitution of canola or olive oils for sunflower oil could attenuate fatty liver status in women with PCOS. Because the effects may depend on the dose of oils consumed, more studies are warranted to determine the proper amounts to achieve the best results.

## Data Availability

Datasets used and/or analyzed during current study are available from corresponding author on reasonable request.

## Abbreviations

PCOS: Polycystic Ovarian Syndrome
CVD: Cardiovascular Diseases
T2D: Type 2 Diabetes
SFAs: Saturated Fatty Acids
MUFAs: Mono-Unsaturated Fatty Acids
PUFAs: Polyunsaturated Fatty Acids
EPA: Eicosapentaenoic Acid
DHA: Docosahexaenoic Acid
ALA: α-Linolenic Acid
IR: Insulin Resistance
TC: Total Cholesterol
HDL: High-Density Lipoprotein
LDL: Low-Density Lipoprotein
TG: Triglyceride
NAFLD: Non-Alcoholic Fatty Liver Disease
BMI: Body Mass Index
OCP: Oral Contraceptive Pill
PPAR-α: Peroxisome proliferator-activated receptor alpha
SREBP-1c: Sterol regulatory element-binding protein 1
HOMA-IR: Homeostatic Model Assessment of Insulin Resistance
SHBG: Sex hormone-binding globulin
FSG: Fasting Serum Glucose
FSI: Fasting Serum Insulin
TNF-α: Tumor Necrosis Factor Alpha
GLUT: Glucose Transporter
